# Pregnancy induced hypertension and mirror syndrome associated with fetal hemoglobin Bart's disease in Thailand: a retrospective cohort study

**DOI:** 10.1016/j.lansea.2026.100764

**Published:** 2026-04-10

**Authors:** Chanane Wanapirak, Suchaya Luewan, Supatra Sirichotiyakul, Fuanglada Tongprasert, Kasemsri Srisupundit, Kuntharee Traisrisilp, Phudit Jatavan, Sirinart Sirilert, Theera Tongsong

**Affiliations:** Department of Obstetrics and Gynecology, Faculty of Medicine, Chiang Mai University, Thailand

**Keywords:** Hemoglobin Bart's disease, Hydrops fetalis, Mirror syndrome, Preeclampsia, Pregnancy-induced hypertension

## Abstract

**Background:**

Pregnancy-induced hypertension (PIH) and mirror syndrome associated with fetal hemoglobin (Hb) Bart's disease have not been well characterized. This study primarily compared the incidence of these conditions in late gestation between affected and low-risk pregnancies, and secondarily assessed differences in other maternal complications.

**Methods:**

A large retrospective cohort study (between 1992 and 2022) was conducted at a single tertiary center. The consecutive singleton pregnancies with fetal Hb Bart's disease diagnosed at ≥24 weeks, considered as delayed diagnosis were recruited into the study group. The control group including low-risk pregnancies with a normal fetus were randomly selected at a 20:1 ratio. Medical records were comprehensively reviewed and validated for the inclusion criteria and pregnancy outcomes. Chi-square test with relative risks as well as Cox proportional hazard ratio were used to compare the main outcomes.

**Findings:**

A total of 142 pregnancies affected by fetal Hb Bart's disease and 2840 controls were included. The incidence of PIH in the study group was 83.8% (89 pregnancies) with a relative risk of 11.33 (95% CI: 9.77–13.15). The subgroup analysis showed markedly higher rates of gestational hypertension, preeclampsia in the study group. The incidence of mirror syndrome was markedly elevated; 70 (49.3%) vs. 2 (0.1%) with a relative risk of 700 (95% CI: 173–2826). The onset of PIH was significantly earlier in the study group. The rare but serious complications were significantly higher in the study group, including a high maternal mortality rate of 2.8%.

**Interpretation:**

Nearly all continuing pregnancies with Hb Bart's disease eventually developed earlier onset and more severe PIH and half developed mirror syndrome with a relative risk of 700 times. Severe morbidity and mortality were also much higher in the affected pregnancies.

**Funding:**

The Chiang Mai University Research Fund CMU-2567.

**Research in context**

**Evidence before this study**

Pregnancy-induced hypertension and mirror syndrome associated with fetal Hb Bart's disease has been documented only rarely, with published evidence limited to case reports and small case series comprising just a few pregnancies

**Added value of this study**

Nearly all continuing pregnancies with Hb Bart's disease eventually developed earlier onset and more severe pregnancy-induced hypertension (PIH), including mirror syndrome with a relative risk of 700. Severe morbidity and mortality were also were increased.

Mirror syndrome represents a form of PIH characterized by maternal edema and is less clinically significant than other serious manifestations, such as HELLP syndrome or eclampsia.

**Implications of all the available evidence**

In cases of Hb Bart's hydrops, clinical attention should be directed more toward identifying severe maternal complications, such as thrombocytopenia, eclampsia, and pulmonary edema, rather than focusing solely on the presence of mirror syndrome.

This study underscores the need for physicians to manage women with mirror syndrome as they would those with PIH with severe features, including close monitoring and prevention of complications such as thrombocytopenia, HELLP syndrome, or eclampsia.

## Introduction

Hemoglobin (Hb) Bart's disease, also known as homozygous alpha-0 thalassemia, is a lethal fetal condition that is particularly common in South and Southeast Asia.^1^, ^2^, ^3^, ^4^ It is one of the most prevalent causes of hydrops fetalis in regions with a high prevalence of thalassemia, such as Thailand.

Currently, with the availability of intensive perinatal care and intrauterine transfusions, an increasing number of patients survive with this condition.^5^, ^6^, ^7^ Nevertheless, these therapies remain limited and are not available in Thailand. When pregnancies with Hb Bart's fetuses are not electively terminated in early gestation or do not receive intrauterine therapy, the disease remains generally fatal to the fetus and the mothers often experience severe complications in late gestation. These include pregnancy-induced hypertension (PIH), mirror syndrome, postpartum hemorrhage associated with a markedly enlarged hydropic placenta, and obstructed labor due to the large hydropic fetus, often resulting in an increased rate of cesarean sections and the psychological impact of carrying a non-viable fetus to term. To date, however, no large cohort study has been published specifically examining pregnancies affected by late-presenting fetal Hb Bart's disease, particularly regarding the association with mirror syndrome or preeclampsia.

In recent years, improved access to obstetric ultrasound has enabled the detection of Hb Bart's disease during the first half of pregnancy, in the pre-hydropic phase, characterized by increased blood flow velocity, cardiac enlargement and functional changes, and placental enlargement etc.,^8^, ^9^, ^10^, ^11^ thereby facilitating early pregnancy termination to prevent the severe complications commonly encountered in late gestation. Moreover, intrauterine therapy has also become available, although only in a very limited number of centers. However, early prenatal diagnosis is not always available in many countries of high, especially in lower income and middle income countries, including in several rural areas of Thailand. Before the widespread use of fetal ultrasound (prior to 1992), delayed diagnosis of Hb Bart's disease was common in Thailand,^2^^,^^3^^,^^12^, ^13^, ^14^ approximately 3–4 pregnancies per 1000 deliveries. To date, though the incidence is very low because of early prenatal diagnosis, of late-presentation still occurs, albeit rarely, and nearly all are referred from rural, low-resource settings. Surprisingly, despite its high incidence and significant maternal morbidity, obstetric complications associated with fetal Hb Bart's disease have been rarely studied. The burden of late-presenting Hb Bart's disease in high-incidence regions, most of which are located in lower-resource settings and lower income and middle income, countries, appears to be underestimated, particularly in South and Southeast Asia.

To the best of our knowledge, PIH and mirror syndrome associated with fetal Hb Bart's disease has been documented only rarely, with published evidence limited to case reports and small case series comprising just a few pregnancies.^15^, ^16^, ^17^ Even systematic reviews and meta-analyses include only a small number of pregnancies, precluding reliable estimation of the true disease burden. Our institution, a tertiary referral center situated in a region with a high incidence of Hb Bart's disease, has had the opportunity to prospectively collect ongoing pregnancies affected by fetal Hb Bart's disease that were referred in late gestation because early diagnosis had been missed. The primary objective of this study was to compare the incidence of PIH and mirror syndrome in late gestation between these pregnancies and low-risk pregnancies with unaffected fetuses. The secondary of objective was to compare the rates of other maternal complications.

## Methods

This retrospective cohort study was conducted at Maharaj Nakorn Chiang Mai Hospital, a tertiary referral center. The study was based on the obstetric database maintained by the Department of Obstetrics and Gynecology, Chiang Mai University. This database has systematically recorded information on pregnant women who delivered at the center from the time of discharge, beginning in 1992 and continuing to the present. All data were digitally entered by the maternal–fetal medicine team. Demographic, clinical, and obstetric outcomes were recorded in the database. Both high-risk and low-risk pregnancies, defined by the presence or absence of pre-existing conditions such as chronic hypertension, heart disease, or thyroid disease, were entered using codes specific to each underlying disease. In addition, pregnancy outcomes, including normal deliveries and complications such as abruptio placentae, fetal distress, or pregnancy-induced hypertension (PIH), were coded to facilitate efficient digital retrieval. Additionally, pregnancies affected by fetal Hb Bart's disease have been prospectively and consecutively entered into the database for over 30 years.

The obstetric database of women who gave birth at our center between 1992 and 2022 was accessed to retrieve all consecutive records of pregnancies affected by fetal Hb Bart's disease. These records were screened to identify pregnancies meeting the following inclusion criteria for the study group: (1) singleton pregnancy; (2) confirmed diagnosis of fetal Hb Bart's disease, based on hemoglobin typing using high-performance liquid chromatography (HPLC) from either fetal blood obtained via cordocentesis or neonatal blood; (3) continuation of pregnancy beyond 24 weeks of gestation, defined as pregnancies with delayed diagnosis; and (4) availability of known final obstetric outcomes.

Women in the control group were randomly selected from digital records of low-risk singleton pregnancies with normal fetuses who delivered at ≥24 weeks of gestation during the same year as the corresponding study cases. Low-risk pregnancies were first extracted from the main database, after which the inclusion and exclusion criteria were applied to validate and compile a new dataset of all eligible low-risk pregnancies for the study. The control group was then randomly selected using the SPSS software, employing the “Random Sample of Cases” function within the “Select Cases” menu. To gain more power, a matching ratio of 20:1 (control to study group) was applied during the selection process.

Exclusion criteria were as follows: (1) multifetal pregnancy; (2) pregnancies complicated by underlying medical conditions such as pregestational diabetes mellitus, renal disease, autoimmune disorders, or chronic hypertension; and (3) incomplete data.

For data extraction, only low-risk pregnancies were initially identified and randomly selected to serve as the control group, as previously described. Demographic and clinical data (e.g., maternal age, parity, underlying medical conditions) and obstetric outcomes (e.g., maternal complications, gestational age at delivery, birth weight, placental weight, mode of delivery) were extracted and validated. Pregnancies with abnormal outcomes, whether from low- or high-risk pregnancies, were identified through digital search and later comprehensively reviewed and validated using full medical records. All pregnancies with of fetal Hb Bart's disease were likewise reviewed, validated, and extracted from complete medical records. The main outcomes were PIH, including gestational hypertension and preeclampsia with or without severe features, mirror syndrome, and other pregnancy complications such as postpartum hemorrhage.

The definitions used in this study are as follows: 1) Pregnancy-induced hypertension (PIH): New-onset maternal hypertension, defined as a blood pressure of ≥140/90 mmHg, occurring after 20 weeks of gestation. 2) Gestational hypertension: PIH without proteinuria. 3) Preeclampsia: PIH accompanied by new-onset proteinuria, defined as a 24-h urine protein excretion of ≥300 mg. Preeclampsia was further subcategorized into pregnancies with and without severe features.^18^ 4) Mirror syndrome: The presence of “triple edema,” including: a) Fetal hydrops: Pathological accumulation of fluid in two or more fetal compartments, such as subcutaneous edema (skin thickness >5 mm), ascites, pleural effusion, or pericardial effusion. b) Placentomegaly: Placental weight exceeding the 90th percentile for the corresponding gestational age. c) Maternal edema: Excessive fluid accumulation in subcutaneous tissue or body cavities, often accompanied by significant weight gain.

### Statistical analysis

The validated data were analyzed using the Statistical Package for the Social Sciences (SPSS), version 26.0 (IBM Corp., Released 2019. IBM SPSS Statistics for Windows, Version 26.0). Continuous data are presented as mean ± standard deviation (SD) or median with interquartile range (IQR), for the normal and non-normal distribution, respectively, categorical data are presented as frequencies and percentages. To compare baseline characteristics and pregnancy outcomes between the study and control groups, categorical variables were analyzed using the Fisher's exact test, Chi-square test, and relative risks (RRs) with 95% confidence intervals (CIs) were calculated. Continuous variables were compared using the unpaired Student's t-test or the Mann–Whitney U test, as appropriate. A Kaplan–Meier analysis was used to illustrate time-to-event (gestational age to development of PIH) data for the development of PIH. A p-value of less than 0.05 was considered statistically significant.

### Ethics statement

Ethical approval was granted by the (Research ID: 0452/Study Code: OBG-2566-0452; Research Ethics Committee Panel 5, Faculty of Medicine, Chiang Mai University, Thailand; approval date: 16 November 2022).

### Role of funding source

The funder of the study had no role in the design of the study, data collection, data analysis, data interpretation, or manuscript writing.

## Results

**Fig. 1:**
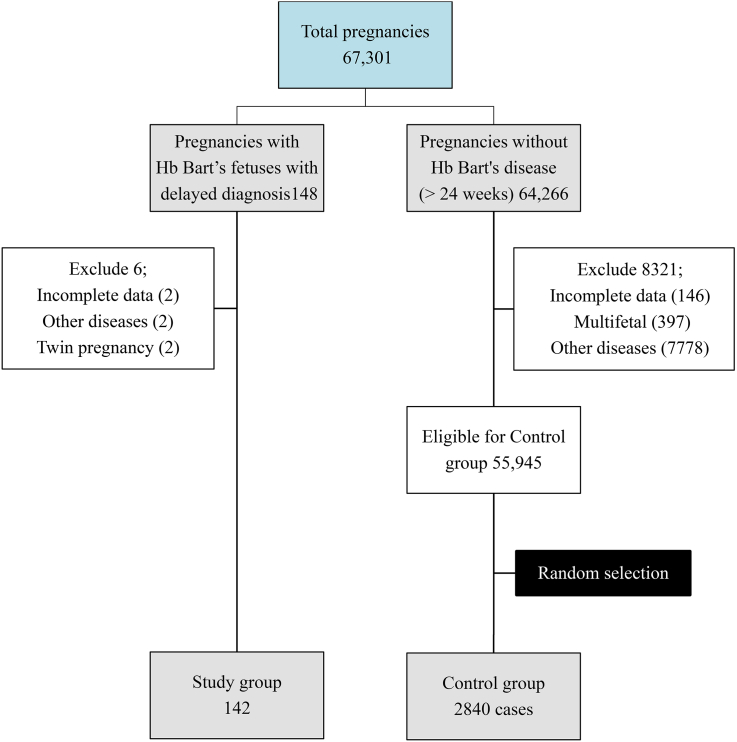
Flowchart of the patient recruitment.

**Fig. 2:**
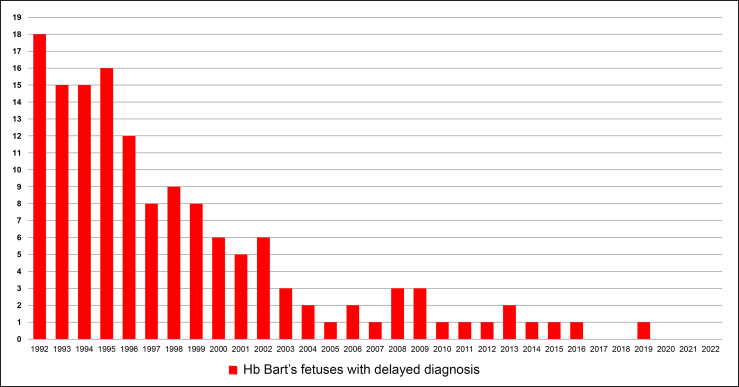
Distribution of the number per year of Hb Bart's fetuses with delayed diagnosis.

During the study period, a total of 148 pregnancies affected by fetal Hb Bart's disease were identified. The incidence of Hb Bart's disease with delayed diagnosis was approximately 0.22% (148 out of 67,301). Six pregnancies were excluded for various reasons, as shown in Fig. 1. The remaining 142 pregnancies were selected for analysis. The majority of these cases (n = 122) were referrals from peripheral hospitals. A total of 2840 low-risk pregnancies with normal fetuses were randomly selected as the control group. Nearly all participants were of Thai nationality and resided in the northern region of Thailand. The largest proportion of pregnancies in the study group occurred during the earlier years of the study period. The incidence demonstrated a clear, continuous decline from 18 pregnancies in 1992 to zero pregnancies in 2022, as illustrated in Fig. 2. All pregnancies after the year 2000 were referred from other centers. Baseline characteristics between the study and control groups were comparable, as summarized in Table 1.

**Table 1:** Baseline characteristics of the two groups.

Characteristics	The study group (N: 142)	The control group (N: 2840)	p-value
Maternal age (years); mean ± SD	27.7 ± 6.3	27.8 ± 6.1	0.889
Gestational age at diagnosis (weeks) in pregnancies with PIH; mean ± SD	29.0 ± 3.5	37.0 ± 2.6	<0.001
Parity			0.972
0	50 (35.2%)	995 (35.0%)	
1	79 (55.6%)	1596 (56.2%)	
2	11 (7.7%)	202 (7.1%)	
3 or higher	2 (1.4%)	46 (1.6%)	
Residency			0.307
Chiang Mai	88 (62.0%)	1919 (67.6%)	
Other parts of northern Thailand	28 (19.7%)	517 (18.2%)	
Others	26 (18.3%)	404 (14.2%)	
Occupation			0.528
Agriculture	4 (2.9%)	110 (4.0%)	
Commercial	6 (4.4%)	233 (8.5%)	
Employee	82 (60.3%)	1452 (52.8%)	
Government Officer	5 (3.7%)	141 (5.1%)	
Private business	2 (1.5%)	81 (2.9%)	
Housewife	32 (23.5%)	639 (23.2%)	
Others	5 (3.7%)	94 (3.4%)	

**Fig. 3:**
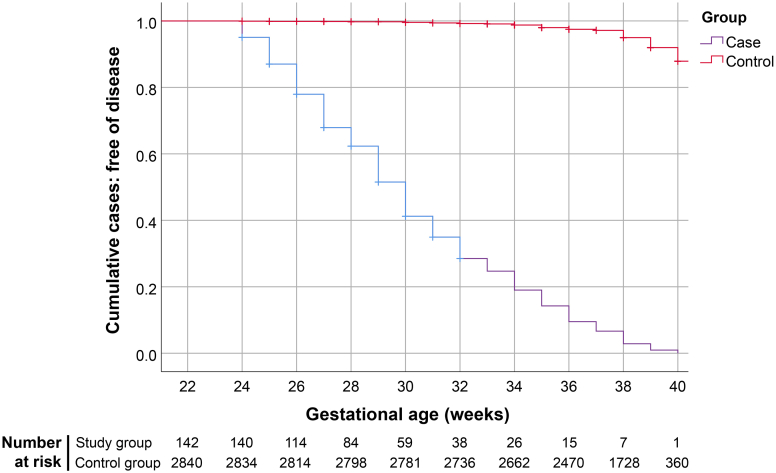
Kaplan–Meier curves of PIH development of the two groups; based on gestational age at the diagnosis; hazard ratio of 71.9 (95% CI: 55.4–93.3).

In comparisons of the main outcomes, the incidence of PIH in the study group was as high as 83.8%, with a relative risk of 11.33 (95% CI: 9.77–13.15). Subgroup analysis also revealed a markedly higher incidence of gestational hypertension and preeclampsia, with and without severe features, in the study group, as presented in Table 2. Similarly, the incidence of mirror syndrome was substantially higher in the study group (49.3% vs. 0.1%), with a relative risk of 700 (95% CI: 173–2826), as shown in Table 2. Notably, in addition to the higher incidence and severity, the gestational age at first diagnosis of PIH was significantly earlier in the study group. The Kaplan–Meier curves demonstrated a significantly lower rate of PIH-free survival in the study group, with a hazard ratio of 71.9 (95% CI: 55.4–93.3) based on Cox proportional-hazards model, as illustrated in Fig. 3. The mean placental weight in affected pregnancies was significantly higher than in the control group, despite the earlier gestational age at delivery. In a subgroup analysis of the control group, the mean placental weight among women with PIH was significantly lower than that among women without PIH (437 vs. 469 g, p < 0.001).

**Table 2:** Comparison of pregnancy outcomes between the two groups.

Characteristics	Study group (N: 142)	Control group (N: 2840)	p-value	Relative risk (95% CI)
Pregnancy-induced hypertension	119 (83.8%)	210 (7.4%)	<0.001	11.33 (9.77–13.15)
Gestational hypertension	49 (34.5%)	82 (2.9%)	<0.001	11.95 (8.75–16.31)
Preeclampsia without severe feature	48 (33.8%)	86 (3.0%)	<0.001	11.16 (8.19–15.22)
Preeclampsia with severe feature	22 (15.5%)	42 (1.5%)	<0.001	10.48 (6.43–17.06)
Mirror syndrome	70 (49.3%)	2 (0.1%)	<0.001	700.00 (173.41–2825.67)
Hydropic placenta	139 (97.9%)	9 (0.3%)	<0.001	308.89 (160.81–593.31)
Gestational age at birth (weeks); mean ± SD	29.4 ± 4.1	38.5 ± 2.6	<0.001	
Birth weight (grams); mean ± SD	2271 ± 1013	2908 ± 526	<0.001	
Placental weight (grams); mean ± SD	1188 ± 516	467 ± 89	<0.001

Rare but serious complications were significantly more frequent in the study group, as shown in Table 3. Most notably, these complications resulted in a high maternal mortality rate of 2.8%, with causes of death including pulmonary edema (n = 1), intracranial hemorrhage (n = 1), and postpartum hemorrhage (n = 2). All maternal deaths occurred in pregnancies referred from peripheral hospitals. All of the neonates were stillbirths with hydropic appearance.

**Table 3:** Comparison of the incidence of severe maternal complications between the two groups.

Characteristics	The study group (N: 142)	The control group (N: 2840)	p-value
Eclampsia	4 (2.8%)	1 (0.0%)	<0.001^a^
Pulmonary edema	5 (3.5%)	2 (0.1%)	<0.001^a^
Intracranial hemorrhage	2 (1.4%)	0 (0.0%)	0.002^a^
HELLP syndrome	5 (3.5%)	3 (0.1%)	<0.001^a^
Postpartum hemorrhage	13 (9.2%)	9 (0.3%)	<0.001^b^
Maternal mortality	4 (2.8%)^c^	0 (0.0%)	<0.001^a^

a Fischer exact test.

b Chi-square test.

c Pulmonary edema (1), intracranial hemorrhage (1), postpartum hemorrhage (2).

## Discussion

The key insights gained from this study are as follows: 1) Pregnancies affected by fetal Hb Bart's disease almost invariably develop PIH in late gestation, including gestational hypertension, preeclampsia, eclampsia, and mirror syndrome, if not terminated earlier. While hydrops fetalis and placentomegaly were present in nearly all pregnancies, mirror syndrome was observed in approximately half. 2) The natural course of PIH associated with fetal Hb Bart's disease tends to be more severe and of earlier onset compared to typical PIH. 3) In continuing pregnancies with fetal Hb Bart's disease, the incidence of serious maternal complications, including postpartum hemorrhage, HELLP syndrome, eclampsia, pulmonary edema, intracranial hemorrhage, and maternal mortality was significantly higher. 4) PIH related to Hb Bart's disease is consistently associated with placentomegaly, resulting in mirror syndrome. This pattern differs markedly from typical PIH, which is generally linked to abnormal placental remodeling and reduced placental size.

It is noteworthy that the incidence of Hb Bart's fetuses with delayed diagnosis was high during the early years of the past three decades but has nearly disappeared in recent years. Therefore, a study of this nature may no longer be feasible in the future, as nearly all pregnancies with Hb Bart's disease are now detected and electively terminated in early gestation due to the widespread implementation of prenatal screening and the availability of obstetric ultrasound in high-incidence regions. Therefore, this study may serve as a historical reference or a legend of fetal Hb Bart's disease, illustrating the natural course and severe maternal consequences of the disease, including serious morbidity and mortality.

Our findings demonstrated a strong association between Hb Bart's hydrops and pregnancy-induced hypertension (PIH), which is likely attributable to placental ischemia. Angiogenesis in the villi, occurring in response to fetal anemia caused by Hb Bart's disease, can increase villous blood flow. This leads to edematous villous stroma, resulting in narrowing of the intervillous space within the placenta.^19^ Consequently, in addition to placental hypoxia induced by fetal anemia, reduced maternal perfusion due to a narrowed intervillous space may further exacerbate placental ischemia. We hypothesize that, the hydropic placenta, resulting from prolonged and profound fetal anemia, develops ischemia or anemic hypoxia and becomes a source of oxidative stress agents released into the maternal circulation,^20^ potentially contributing to the development of preeclampsia. As a result, microangiopathies occur in all over the body, leading to various manifestations of PIH or preeclampsia. Our previous observations indicate that placentas from pregnancies affected by fetal Hb Bart's disease release oxidative stress biomarkers into the maternal circulation even in early pregnancy, prior to the clinical onset of preeclampsia.^20^ Taken together, these findings suggest that oxidative stress originating from the ischemic placenta gradually increases with advancing gestational age, contributing to a progressively severe clinical course, ranging from subtle or subclinical manifestations in early pregnancy to life-threatening preeclampsia in the third trimester.

Typically, preeclampsia is strongly associated with placental ischemia, commonly resulting from abnormal placental remodeling during early gestation, referred to as placental insufficiency. In such situations, the placenta is usually small, as observed in preeclamptic pregnancies within the control group. In contrast, preeclampsia associated with fetal Hb Bart's disease is almost always accompanied by an enlarged or hydropic placenta. We hypothesize that preeclampsia may result from placental ischemia caused either by hydropic/anemic villi, as seen in fetal Hb Bart's disease, or by abnormal placentation, as seen in placental insufficiency. Although these two entities differ markedly in their gross placental appearance, they share a common pathological pathway, placental hypoxia leading to the release of oxidative stress agents into the maternal circulation. As previously described, typical preeclampsia resulting from abnormal placentation follows a two-stage pathogenesis: the first stage involves localized placental pathology in early pregnancy, and the second stage occurs when oxidative stress agents enter the maternal circulation in increasing amounts, triggering the clinical manifestations of preeclampsia.^21^ A similar process is believed to occur in pregnancies complicated by fetal Hb Bart's disease, where placental ischemia subtly begins in the early phase.^20^ At this early stage, the placenta is already enlarged,^22^ likely due to fetal hypervolemia secondary to anemia.^23^ This volume overload contributes to fluid leakage into the interstitial space, leading to hydropic changes, including within the villi, and ultimately placental enlargement. Placental ischemia in fetal Hb Bart's disease is likely caused by a combination of anemic hypoxia and poor maternal perfusion resulting from narrowing of the intervillous space due to hydropic villi.^19^ Based on these observations, we it may be worthwhile to consider PIH as two distinct types: (1) small placenta type, caused by abnormal placental remodeling in early gestation (as seen in typical PIH), and (2) enlarged placenta type, caused by fetal anemic hypoxia (as seen in pregnancies with fetal Hb Bart's disease). Both types have the common pathogenesis in terms of placental ischemia but different in the causes of ischemia. Because the enlarged-placenta type is driven by fetal anemia, it is potentially treatable with intrauterine therapy, and mirror syndrome may be completely reversible, as demonstrated in two pregnancies reported by Chimenea et al.^24^ Additionally, PIH or mirror syndrome associated with fetal Hb Bart's disease can be prevented through regular intrauterine transfusions, as demonstrated where pregnancy was successfully prolonged to allow for definitive postnatal treatment.^5^, ^6^, ^7^ In contrast, the small-placenta type is associated with hypoxia caused by abnormal placentation and is generally untreatable, apart from termination of pregnancy.

It is evident that continuing a pregnancy affected by fetal Hb Bart's disease markedly increases the risk of PIH, according to standard diagnostic criteria, across all levels of severity. Given that the placenta in all affected hydropic fetuses is markedly enlarged, and considering that maternal edema is a common feature of PIH due to fluid leakage caused by systemic microangiopathies, these pregnancies are highly predisposed to meeting the diagnostic criteria for mirror syndrome. In this study, pregnancies affected by fetal Hb Bart's disease exhibited an elevated risk of mirror syndrome, with a relative risk approximately 700 times greater than that of the control group. These findings support the notion that mirror syndrome may be considered a variant of PIH, akin to HELLP syndrome or eclampsia However, due to its diagnostic definition, mirror syndrome may be considered a specific subtype of PIH associated with a hydropic placenta. Importantly, the hydropic placenta, rather than the hydropic fetus, appears to be the primary driver of maternal PIH. Supporting this-, hydrops fetalis caused by Turner syndrome, which typically does not involve placentomegaly, is rarely associated with mirror syndrome.^25^

The incidence of mirror syndrome in our study is higher than that reported in previous studies,^16^^,^^17^^,^^24^^,^^26^, ^27^, ^28^, ^29^ which range from 5% to 37% among pregnancies complicated by fetal hydrops due to various conditions such as Rh isoimmunization, parvovirus B19 infection, and placental chorioangioma. This discrepancy is likely attributable to the greater severity of disease in our cohort, as fetal Hb Bart's disease is generally fatal to the fetus and is typically associated with marked placental enlargement, with placental weights commonly reaching 1–2 kg in late gestation.

Our findings demonstrate that fetal and placental hydrops may give rise to a spectrum of clinical manifestations ranging from gestational hypertension to preeclampsia with severe features or eclampsia, similar to those observed in classic PIH not associated with fetal hydrops. While nearly all affected pregnancies developed PIH, some developed mirror syndrome without obvious clinical maternal edema. Therefore, it is reasonable to suggest that mirror syndrome shares a similar underlying pathophysiology as PIH. Importantly, clinical manifestations of mirror syndrome closely resemble those of classic PIH unrelated to fetal hydrops.

In pregnancies with history of fetal hydrops, clinical attention should be directed more toward identifying severe maternal complications, such as thrombocytopenia, eclampsia, and pulmonary edema, rather than focusing solely on the presence of mirror syndrome. From our perspective, mirror syndrome represents a form of PIH characterized by maternal edema and is less clinically significant than other serious manifestations, such as HELLP syndrome or eclampsia. This concept underscores the need for physicians to manage women with mirror syndrome as they would those with PIH with severe features, including close monitoring and prevention of complications such as thrombocytopenia, HELLP syndrome, or eclampsia.

We would also like to highlight the limitations of the study. Weaknesses of this study include: 1) The retrospective nature of the study, which may compromise the reliability of certain parameters based on subjective assessment, such as the diagnosis of maternal edema. 2) The long study period, which could introduce temporal variability; however, this is unlikely to have significantly influenced the natural course of the disease or the study's overall conclusions. 3) The use of Hb Bart's disease as a study model, which, although representing a severe form of fetal anemia, may not fully reflect the pathophysiological features of fetal anemia resulting from other etiologies. 4) Our findings are based on data from a single center, which may limit their generalizability to other settings.

Strengths of this study include: 1) The largest sample size to date, with high homogeneity in the etiology of fetal anemia and an adequately sized control group. This contrasts with a recent large systematic review of 12 studies,^15^ which included 82 pregnancies with mirror syndrome with heterogeneous causes and varying severity of fetal hydrops, making it difficult to draw definitive conclusions. 2) Although the study was retrospective in design, all histories were thoroughly reviewed using complete medical records, ensuring the accuracy and reliability of the collected data.

In conclusion, nearly all continuing pregnancies affected by Hb Bart's disease developed earlier-onset and more severe pregnancy-induced hypertension (PIH), with approximately half also meeting the criteria for mirror syndrome, demonstrating a relative risk increase of 700-fold. Severe maternal morbidity and mortality were significantly higher in the affected group. Based on these findings, we propose that PIH be classified into two subtypes: small placenta PIH and large placenta PIH. The term mirror syndrome may be reconsidered, as it may not adequately reflect the underlying pathophysiology or clinical significance for maternal health.

## Contributors

Database development: CW, TT.

Conceptualization: CW, SL, SiS, TT; Data collection: CW, SS, FT, KS, SL, KT, PJ, SiS, TT.

Validation: CW, SS, FT, KS, SL, KT, PJ, SiS, TT.

Manuscript writing: CW, TT, SiS, TT.

Manuscript editing: SS, FT, KS, KT, PJ.

Final approval: CW, SS, FT, KS, SL, KT, PJ, SiS, TT.

## Data sharing statement

The datasets analyzed during the current study are available from the corresponding author upon reasonable request.

## Declaration of interests

The authors declare that they have no competing interests.
